# Working the edges of Posthuman disability studies: theorising with disabled young people with life‐limiting impairments

**DOI:** 10.1111/1467-9566.12962

**Published:** 2019-06-07

**Authors:** Kirsty Liddiard, Sally Whitney, Katy Evans, Lucy Watts, Emma Vogelmann, Ruth Spurr, Carrie Aimes, Katherine Runswick‐Cole, Dan Goodley

**Affiliations:** ^1^ School of Education and iHuman University of Sheffield Sheffield UK

**Keywords:** disability, youth, palliative, coproduction, research

## Abstract

This paper is built upon an assumption: that social theory can be generated through a meaningful engagement with a co‐researcher group of disabled young people. Our co‐researchers are theoretical provocateurs and theorists in their own right who, through their activism and writing, are challenging us to reconsider the meaning of life, death and disability. Their work on our funded Economic and Social Research Council (ESRC) project has enabled us to consider the promise and potential of humanist and posthuman epistemologies, theories, methodologies, interventions and activisms. The paper introduces the research, the authors of this paper (academics and co‐researchers) and then explores three layers of analysis that work the edges of posthuman thinking; sovereign and assembled selves; affects and desires; mourning and affirmation. We conclude by asserting that as a research team we are engaging with a DisHuman approach to theory and activism: one that blends the pragmatics of humanism with posthuman possibilities.

## Introduction

This paper is built upon an assumption: that social theory can be generated through a meaningful engagement with a group of disabled young people. This paper understands co‐researchers of disabled young people as theorists and, crucially, promotes them as theoretical provocateurs. It is not simply the case that young people would explicitly identify themselves through the language or concepts of theory. Nor would they necessarily identify as theoreticians. Rather, our work with them has demonstrated the possibilities that they offer us to think about the world in different ways, which we offer here as but one definition of theory.

Our interest in posthuman disability studies has been heightened through our work on the research project, *Life, Death, Disability and the Human: Living Life to the Fullest* (hereby Living Life to the Fullest). Our study takes place in the UK with disabled young co‐researchers via a Co‐researcher Collective ‐ currently five disabled young women aged 19‐30 who identify as living with ‘life limiting’ and ‘life‐threatening impairments’ (hereby LL/LTIs) ‐ from across the country. Through engagement with their narratives and the arts, Living Life to the Fullest^1^ seeks to forge new understandings of the lives, hopes, desires and contributions of children and young people with LL/LTIs. Knowledge production about and around the lives of those with LL/LTIs rarely comes from disabled children and young people themselves. This knowledge tends to be medicalised, individualised and pathologised: reflecting the prognoses and imagined futures of others such as associated practitioners, service providers and professionals. Our work seeks to bring in emic accounts and expertise from disabled young people with LL/LTIs working alongside academics to constitute a research collective.

The idea for a Co‐Researcher Collective originated through early research and impact planning workshops with disabled young people and their families when writing the bid for funding. Our emphasis was to work in ways that ensured the inception of the research process accessibly drew in a shared distribution of responsibility from the outset. We desired to contest the orthodox, dis/ableist and elitist ways in which research‐funding bids are generated (by academics for academics) and how funding itself is allocated (which has historically privileged university researchers and sidelined non‐academic researchers, NGOs and those researching outside of traditional academic contexts) (Runswick‐Cole *et al*. 2017). Young people, their families and our community partner organisations stressed that disabled young people should have a significant role in the project as researchers and leaders (see Kitchen 2000).

Co‐researchers’ contributions have been foundational to the empirical and theoretical development of the project. Co‐Researchers have (i) supported research design and planning; (ii) co‐written interview schedules and carried out data collection with fellow young people through qualitative semi‐structured interviews via new social technologies; (iii) recruited participants and developed relationships with impact partners via their own networks; (iv) co‐led the research process and collaborative analysis through meetings, workshops and as contributors to a broader Research Management Team of academics, parents, and representatives and partners from disability and arts organisations; (v) written blogs and made a short documentary film (The Co‐Researcher Collective, 2018); (vi) presented at conferences; and (vii) co‐written articles for publication (Liddiard *et al*. 2018) (fittingly, we have co‐authored and revised this article through online discussions via Skype and the use of a shared Google document). These critical contributions have meant that we are co‐producing theoretical knowledges of the lives of disabled children and young people with LL/LTIs and their families.

Let us start by addressing the elephant in the room; the apparently contradictory nature of the work we seek out to do that is embodied in the title of our paper. Posthuman approaches, as we shall explain, emerge as a response to the fixity of classical, modernist and humanist conceptions of the human. While these humanist formations are predicated upon some kind of bounded, rational, autonomous and sovereign human subject, the posthuman condition suggests something more expansive, relational and nomadic. And yet, in our title, we define our co‐researchers as disabled young people with life‐limiting impairments. This label implies that lives are limited both in terms of length and quality. In adopting such medicalising labels we inevitably draw in a priori conceptions of a lacking human subject living a short life that grates with everyday normative ideas of what constitutes a good life (read; a life long‐lived). This version of the human sits uneasily with the more affirmative offerings of the posthuman. Also, the young people with whom we are co‐producing the research fundamentally contest this version. This is but one contradiction. Moreover, our title pulls in disability studies: an interdisciplinary field of inquiry, scholarship and activism that seeks to quash damaging pathological discourses of disability to offer more socio‐cultural conceptions (see for an overview Goodley *et al*. 2014, Goodley 2016, Liddiard 2018). ‘Life‐limiting’ appears to concede these young people to medicalisation and is at odds with the transformative offerings of critical disability studies. Nevertheless, our concern is with the lives and aspirations of these young people whose identities have been partially defined through the rhetoric of life‐limiting. So, how might we bring together these contradictory elements in order to offer theoretical alliance to these young people?

We suggest that assembling these terms together offers us affirmative possibilities to contribute to the fields of critical disability studies and medical sociology. We should not feel blocked by contradiction. Instead, a posthuman perspective affirms that we are rooted but we flow: encouraging ‘us to recognise the intersections between mobility, multiple identities, and ethical belonging and accountability’ (Braidotti and Regan 2017: 212). This idea anticipates the concluding elements of this paper when we bring in a DisHuman perspective (Goodley *et al*. 2014): a contrary position that is occupied by disability that disavows the humanist human category. Being human and becoming posthuman characterises not only the DisHuman position of our co‐researchers but also the contemporary moment occupied by us all in times associated with austerity and precarity. In the next section, we expand further on our theoretical preoccupations and ambitions.

### The centrality of humanism and the emergence of posthuman thinking

The last decade has witnessed a coming of age of the posthuman condition. Many key proponents of posthuman theory are women and feminists (Karen Barad, Rosi Braidotti and Donna Haraway) and connections have been made with postcolonial, queer, black, trans, indigenous and, significantly for us, critical disability studies scholarship (e.g. Flynn 2017, Goodley *et al*. 2014, Reeve 2012, Vandekinderen and Roets 2016). Our paper builds on a strong tradition of scholarship that seeks dialogue between disability studies and medical sociology (e.g. Shakespeare and Watson 2001, Thomas 1999, 2007). Specifically, we are interested in recognising and challenging humanism, which has been a central philosophy since the European renaissance of the 14th, 15th and 16th Centuries (and in many cases has underpinned medical sociology and disability studies’ conceptualisations of the human). Humanism will be ever associated with the birth of a citizenry deemed able to speak and write with eloquence and clarity. Whereas previous centuries, at least in Western and European contexts, tied human activity to the power of the monarchy and the truths of religion, humanism heralded in a particular period of the *holocene*: ‘a geological epoch during which Homo Sapiens flourished’ (Castree 2015: 66). Rationality, science and democracy encapsulated a specific kind of human activity in the world. In short, given the right conditions, this worldview posits that human beings are capable of maintaining their species‐dominance through the offerings of rational thought, self‐governance and progress towards objective knowledge. Humanism has not gained dominance without human, non‐human and environmental collateral: creating the *anthropocene*: ‘a new era marked by unprecedented rates of human activity on the planet and planetary ecosystems’ (Wallin 2017: 1099).

To argue that we live in *posthuman times* acknowledges the impact human beings have had on the world and on themselves. For Braidotti (2013: 159), the ‘this new knowing subject is a complex assemblage of human and non‐human, planetary and cosmic, given and manufactured, which requires major re‐adjustments in our way of thinking’ (Braidotti 2013: 159). The posthuman citizen is a pragmatic term acknowledging the amalgamation of biology/technology that is ‘deeply intertwined with the planetary and the cosmos; intimately and complexly entangled in relationships with other humans and non‐humans; a globalised entity of virtually infinite proportions’ (Braidotti 2013: 37). The great ‘emancipatory movements of postmodernity’ (Braidotti 2013: 37) – associated with class, animal, sexuality, race, global, sexual, gender, trans‐national and queer politics – have their ‘fires stoked by structural others such as the pro‐environment, anti‐nuclear, anti‐globalisation’ and, we would add, disability movements (Ibid.). Unlike many of her posthuman peers, Braidotti (2013: 146) has been quick to acknowledge that ‘disability studies is almost emblematic of the posthuman predicament’ combining ‘the critique of normative bodily models with the advocacy of new, creative models of embodiment’ (Braidotti 2013: 146). We welcome disability becoming an integral part of contemporary posthuman thinking (e.g. Braidotti and Hlavajova 2018). We note, however, that disabled people have been historically excluded from various iterations of humanist thinking ‐ this tension is a recurring thread of this article.

## Our study and writing collaboratively

The Co‐Researcher Collective is enabling a radical revision of the didactic ways in which research into disabled young people's lives is typically carried out. Our co‐researchers include Lucy Watts MBE, Emma Vogelmann, Katy Evans, Sally Whitney, Carrie Aimes and Ruth Spurr. Notably, our co‐production politics regard co‐researchers as leaders who have ‘an alternative, legitimate expertise to that of academic researchers’ (Nind *et al*. 2012: 660), countering common methods of co‐production with young people that centre on tokenism and restrictions around which parts of the process they can contribute to effectively (see Coad and Lewis 2004).

Our project participation and leadership is shaped and adapted to fit around the needs and desires of young people. Nearly all of The Co‐Researcher Collective takes place online across multiple virtual environments and social media platforms ‐ we connect daily through a lively WhatsApp project thread, Skype, email, Twitter and a closed Facebook group. This been an inclusive and accessible means of practicing research with young people with significant medical, care and access needs due to online spaces being typically more malleable to different embodiments, capabilities and bodily functions (see Liddiard 2013, Seymour 2001). However, it has also gone on to further mediate the ways in which we have undertaken key aspects of the project to proffer new forms of inquiry: data collection; collaborative analysis; project co‐leadership; and dissemination of the process and making impact (see Liddiard *et al*. 2018). Co‐researching has impacted on our shared theoretical thinking in relation to life, death and disability. While co‐researchers have provided thoughtful counsel in relation to the methods and methodologies of the project, they have also offered their own perspectives through blogs and other multimedia resources that have profound impacts on the theoretical work of our study. We draw on four pieces in this paper to demonstrate the impact of our co‐researchers on our thinking as a research team:

**Resource 1 ‐ Presentation to canine partners (presentation) ‐** In this presentation, given to Canine Partners [an organisation that provides assistance animals to people with specific health and access needs], co‐researcher Sally Whitney discusses her relationship with her assistance dog, Ethan (Whitney 2018).
**Resource 2** 2 – **Co‐researcher voices: speaking out (project blog post) ‐** This blog post draws on an article published in Huffpost, by co‐researcher Emma Vogelmann, about the Cambridge Analytica privacy scandal and the meaning of social media to disabled young people. Emma offers a reflexive account of her own decision to leave Facebook in protest to Facebook's actions (Vogelmann 2018).
**Resource 3** 3 ‐ **Planning for the end of my life, aged just 17, made me live a life that ensured i wasn't forgotten (project blog post)** – This is a bespoke blog post written by Lucy Watts MBE for our project website about the meanings of end‐of‐life planning as a young disabled woman (Watts 2017).
**Resource 4** 4 ‐ **Exploring the world side by side ‐ (project blog post) –** This blog post was written from a conference paper given at a symposium hosted by the Institute for the Study of the Human (iHuman).^5^ Project researcher Kirsty Liddiard and Lucy Watts MBE originally co‐wrote the paper through conversation (Watts and Liddiard 2017).

Shaw (2012: 42) argues that online blogs have the potential to contribute to what has been defined as ‘discursive activism’; that is ‘speech or texts that seek to challenge opposing discourses by exposing power relations within these discourses, denaturalising what appears natural’. Virtual forms of activism are especially powerful for disabled people, and have surged in an austerity age (Goodley *et al*. 2019). The virtual world is argued to offer new forms of citizenship due to the Internet providing more accessible avenues for participation, communication, education, entertainment, and employment than in the ‘real world’ where significant barriers forcefully prevail (Seymour 2001). As our co‐researcher Emma Vogelmann articulates:

Having a social media presence is how I started my activism career. The day I wrote my first blog on the attitudes and discrimination I encountered as a disabled student, I posted on Facebook. When I realised I could reach an even wider audience by having a larger presence, I jumped at the chance to make my voice as widely heard as possible (Vogelmann 2018: np).

Crucially, the internet has engendered a ‘democratising’ effect, especially within highly sensitive topics of debate such as those associated with life, death and dying. However, we know that many disabled people are still digitally excluded (especially people with the label of intellectual disability and those with visual impairments). Other forms of marginalisation, including poverty and gender also intersect with disability to produce digital marginalisation. Notwithstanding these exclusions, we argue that these resources do things in the world in their own right and we want to explore how they articulate humanist and posthuman positions.

This paper was written collaboratively. Initially, project researchers Dan Goodley, Katherine Runswick‐Cole and Kirsty Liddiard developed a draft that we then shared with Emma, Katy, Lucy, Sally, Carrie and Ruth. Co‐researchers wrote into the piece, making additions and changes to the analysis, expanding on the blog posts and offering new information. Our co‐researchers are engaging in forms of online empirical enquiry and participatory co‐production that are at the very epicentre posthuman by design: emphasising relationality, blurring voices and ontologies and merging digital contributions from disparate physical and geographic locations.

## Analysis

Our analysis of the resources listed above adopts a broad thematic analysis, where we tease out recurring themes across the different blogs and presentations. We do want to acknowledge, however, that our interests in post/human philosophy guided how we read the texts and produced the thematic categories elaborated below: (i) Sovereign and assembled selves; (ii) affect and desires; and (iii) mourning and affirmation. We take heart from other researchers who have explicitly written about the ways in which their own theoretical preoccupations have directly impacted upon their analytical writing. Some readers might feel we are shoehorning the data into pre‐prepared categories of analysis. Our justification is three‐fold. First, we believe that we have found a resonance between some of the literature around post/human theories and the theoretical contributions of our co‐researchers. Second, that we have written this paper collaboratively at least has allowed us to explain, debate and clarify our developing ideas with one another in relation to the utilisation of theory on and upon data (and the relative dilemmas this raises). Third, we share a conviction to the belief that analysis is never theory‐free, nor neutral nor simplistically inductive. We take issue with the mistaken idea that data and data alone drive analysis. We are theoretical animals. And so are our co‐researchers. In ensuing analysis, then, we tease out three themes that connect specifically with the lives of disabled young people with LL/LTIs.

### Sovereign and assembled selves

Humans beings often define themselves in terms of their abilities: ‘the ability to speak, to act, to create, to think, to feel, to be self‐aware, to exercise free will’ (Saur and Sidorkin 2018: 6). This is humanism and those that cannot enact these abilities risk being excluded: for ‘failure to contribute to the reciprocal economy of the able’ (Saur and Sidorkin 2018: 7). Historically, humanism has embraced some humans and constituted others as sub‐human or in‐human through slavery, categorisation, institutionalisation, demonisation and marginalisation. As avid proponents of some of the contributions of posthuman theory we worry about the divisive qualities of humanism: the rejection of those human beings who fail to reach its exalted standards of human worth. We are concerned with the ways in which, under this regime, those that are valued subjects tend to coalesce around the same kinds of human. ‘Normative humanity’, Braidotti (2013: 24) writes, ‘is very much a male of the species: it is a he’. Moreover, ‘he is white, European, handsome and able‐bodied’ (Braidotti 2013: 24). Within critical disability studies we would understand humanism as a key element of ableism (Campbell 2009). Ableism denotes broad cultural logics of autonomy, self‐sufficiency and independence. We would want to consider ability (and the craving of ability tied up within ableism) in similar ways. Neoliberal‐ableism is the elision of national economic independence with an individual and cultural celebration of autonomy (Goodley 2014). This particular affect economy ties individual and national progress to self‐determination and, by virtue of this, associates happiness with self‐reliance. Hence, while people with physical, sensory and cognitive impairments risk experiencing disablism, all individuals of contemporary society are imperilled by the practices of ableism. In our culture these values are framed with reference to humanist ideals that equate the subject with rationality, consciousness, moral and cognitive universalism.

Yet, we have found through our work with co‐researchers that humanism remains a key guiding philosophy associated with finding oneself a place in the world (Goodley *et al*. 2014). This is hardly surprising when much of what we understand as cultural politics is under‐girded by humanist and ableist ideas. A number of our co‐researchers use the language of humanism in order to affirm their own sense of self:

In February 2012, I was diagnosed with another rare genetic condition – Ehlers‐Danlos syndrome. There was no cure. In my previous healthy life, everything that I had wanted I was able to achieve by pushing hard and working at things until I got to the place I wanted to be. I adopted the same methodology to overcoming my illness. I had to decide how I was going to get to Edinburgh to study medicine. I think I still thought that I could push through and do things on my own, through hard work, as I'd always done.’ (Whitney 2018).

Lucy Watts MBE is a 24‐year‐old woman: an activist, campaigner, writer and charity worker. In 2016, Lucy was awarded an MBE for her services to young disabled people (Watts and Liddiard, 2018: np).

As disabled women ‐ historically written out of humanist discourses ‐ their claiming of their individual worth is entirely understandable. They demand a place at the humanist table. However, when probed further, what is interesting is that our co‐researchers do not simply stay within a humanist realm. While emphasising their human(ist) characteristics, our co‐researchers have also alerted us to the potentialities invested in their nested relationships with other humans and non‐humans: as assembled selves connected with and to non‐human others. A posthuman condition entails the ‘displacement of anthropocentrism and the recognition of trans‐species solidarity’ (Braidotti 2013: 67). Connections between humans and animals provide vital interconnections; positing a ‘qualitative shift of relationship away from speciesism and towards an ethical appreciation of what bodies (human, animal, others) can do’ (Braidotti 2013: 71). Our co‐researchers understand this blurring of human‐animal bodies, and they talk in very posthuman ways. In Resource 1, Sally discusses the impact her canine companion has had on her life:

Ethan brings a freedom to the oppressiveness that requiring 24 hour care can bring. He allows me an independence in the home that I otherwise wouldn't have. My carers do all of the required tasks for me but achieving them on our own, between me and Ethan, brings a fabulous sense of freedom. Ethan has proved invaluable at keeping me safe and allows me to be in rooms on my own without the need for constant checks to make sure I'm OK. He can use a bell to ring carers, he runs to get help from a carer by nudging them and leading them to me. He is able to recognise symptoms of a seizure or collapse before I'm aware of them and will alert me to them and then get help. He is trained to lie next to me while fitting. I no longer have any time when I feel alone or isolated. I always have my trusty sidekick who loves me and believes in me no matter what. His unconditional love and constant desire for cuddles is a treatment in itself! Working towards new tasks and awards with Ethan has gone a long way to giving me back that need for industry and a sense of purpose (Whitney 2018).

We consider co‐researchers’ relationships with non‐human animals not just as a technology for enabling (humanist) desires but as a posthuman intimacy that offers expansive ways for living. And intriguingly, Sally considers the ways in which Ethan's presence impacted hugely on her relationships with other humans:

With Ethan by my side, the topic of conversation is not around my health but about Ethan, how handsome he is and what tasks he can do. Discussion shifted from the negative aspects of my ill health to the incredibly positive asset of having an assistance dog. People stop me in the street to comment on Ethan as opposed to how sad it is to see a ‘young, attractive’ woman in a wheelchair. I had cripplingly low self‐esteem, carved from the punitive treatment and disbelief I had received in hospital. Ethan challenged me to believe that I could be of worth to someone else. It was this realisation that led me to have the confidence to start dating and beginning to see myself as a ‘desirable’ individual (Whitney 2018).

We have returned full circle back to the importance of the being recognised as an valued individual human being (a humanist recognition). Similar insights are provided by Lucy and Emma:

Molly carries out a range of practical tasks with and for Lucy. Things like ‘picking up dropped items, undressing her, grabbing the post, loading and unloading the washing machine, pressing lift buttons, carrying items and paying cashiers’ (Watts and Liddiard 2018, np).

Prior to Molly, people were too scared to talk to me in fear of saying the wrong thing. I felt invisible, because people didn't acknowledge my existence, except from staring at me from a distance. With Molly, however, people were coming up to me and instigating conversation. From the extremely shy girl I was, I was blossoming into someone who felt able to talk to others, to hold conversations with people I'd never met. I felt a part of society again, and that was all down to Molly (Watts and Liddiard 2018, np).

I don't want to feel socially isolated so I am keeping some social media accounts but deleting one that I morally disagree with. This is my choice and you can make yours (Vogelmann 2018: np).

It is through these human‐animal‐technology assemblages that our co‐researchers get a place at the humanist table. However, subjectivity is not restricted to bound individuals, it is also a ‘co‐operative trans‐species effort… that takes place transversally, in‐between nature/technology; male/female; black/white; local/global; present/past – in assemblages that flow across and displace the binaries’ (Braidotti 2018: 2). Disability illuminates the tension between our sovereign and assembled selves; something that we all experience in different ways across the lifecourse. One of the gifts of disability is its disruptive potential to acknowledge such tensions and celebrate our interdependencies. Our co‐researchers work the edges of humanism and the offerings of posthuman alternatives.

### Affects and desires

We have previously argued that the affective turn in social theory takes on a particular resonance for us when we are working with our co‐researchers. We continue to sit with this position and recognise that to speak and feel of life and death is always culturally mediated and party to various formations of embodied and affective performances. At the heart of affect is desire. Gorton (2008: 18) writes that: ‘desire has been understood as both an emotion and an affect, as a drive, and as the essence of human subjectivity’. This definition beautifully captures the contradictory and oppositional ways in which different understandings of affect and desire sit in relation to one another. Desire tends to be understood in terms of what we lack; we are affectively attached to something or somebody on the basis that that object or subject will satisfy and sate. This is the model of desire found in psychoanalysis, captured by Lacan, in which the human being is driven by this psychical search for plenitude. This model of desire is also a dominant one of contemporary capitalism. As Ahmed (2004) has articulated, desire is articulated through the power and circulation of various affect economies that shape the objects of desire. As we argue, ‘nurture, affection and care are shaped through complex political, cultural and social economies’ (Goodley *et al*. 2018: 200):

The four walls of my bedroom had become my prison; but my dog had set me free (Watts, 2018, np).

I was unable to sit up and eat since the movement triggered a seizure, and I lost a lot of weight, dropping to 5st. Bedbound, weak and exhausted, I felt I was losing everything. The following years would see me yo‐yoing between feeling well enough to re‐apply to medical school and start at university and constantly visiting consultants for tests, further diagnoses and surgeries. I tried to maintain the identity of a young student who was dealing with health problems and rejected the notion that I had incurable diseases and life‐threatening impairments. All I did know was that it was an incredibly lonely existence. I felt like I did not have anyone who I could relate to who was in or understood my situation. I thought that if I opened up about my situation it would alienate me further (Whitney 2018).

Faced with a disabling society, our co‐researchers illuminate the powerful ways in which affect economies associated with health and mobility impinge upon affects and desires:

Being with him felt so natural and despite Ed admitting he wasn't really a dog person, Ethan quickly won him round! From then on, our relationship blossomed (Whitney 2018, np).

As Gorton (2008: 31) writes, a posthuman conceptualisation of desire is ‘figured in terms of what it does and how it moves people’. Posthuman articulations of desire produce ‘new ontologies of the decentred subject: this is a post‐human version of a non‐unitary, impersonal and post‐identitarian subject that foregrounds the more‐than‐human’ (Renold and Ivinson 2014: 364). Thus ‘assemblages’ can be made up of all manner of matter: corporeal, technological, mechanical, virtual, discursive and imaginary, that carry affective charges (Renold and Ivinson 2014: 364). Here desire is not about lack, but is productive and a way of making connections with other non/humans. This is not a desire that is oriented towards or directed by something (i.e. the imaginary) and does not sit outside the social, in fantasy. Instead, desire is productive; it produces the real (Renold and Ivinson 2014: 373), as we see in Sally's words here:

While I don't know what my future holds, I'm living in the moment and enjoying every second. I am incredibly grateful to be blessed with my assistance dog Ethan and husband Ed (Whitney 2018, np).

This extract contains remnants of both a productive element of desire (‘living in the moment’) and a more humanist desire for connection with others. Similarly, reflecting on the meaning of Molly in her life and to her future, hope and happiness, Lucy states:

I owe her [Molly] an awful lot. My happiness, my purpose, my positivity, my zest for life, my fulfilment, my work, my life; I'd go so far as saying my existence. She's changed my life beyond all recognition, but she's saved it too – in more ways than one (Watts and Liddiard 2018, np)

Hope and happiness is very much at the heart of an affirmative model of desire; a model that may not have definitive outcomes nor aspirations but is one that celebrates one's connections in the world. In these precarious times, we all struggle with what the future holds, connection with each other, and with animals, seems to offer an affirmative response to precarity. At the same time, it is impossible and undesirable to give up on a humanist desire of that which is lacking. The drive to obtain those elements of everyday life and participation enjoyed by others – and often denied to disabled people – is very much at the heart of the humanism of disability politics. What we are finding, however, is that for our co‐researchers the story does not stop there. Instead, they are engaged in a host of humanist and posthuman modes of desire and desiring (a point we elaborate later in relation to our DisHuman position).

### Mourning and affirmation

A predominance of social theory, especially of a deconstructionist and poststructuralist bent, works from a melancholic thanatopolitics, where the human condition is conceived of as a broken entity in need of repair. Braidotti (2010: 43) maintains that this ‘school of thought stresses the vulnerability and passivity of precarious life‐forms and the importance of mourning’. Unsurprisingly our co‐researchers are more than aware of their precarity:

I was born with health problems that went undiagnosed and unconnected despite seeing many professionals over the years. I deteriorated throughout childhood, living an active life despite my struggles, and hiding what was going on as best I could. When I got to the age of nine things started to deteriorate, from age 11 I had physiotherapy and despite being active riding horses and working at the stables, my deterioration continued until my body gave up in January 2008. I became disabled. My condition continued progressing, my muscles were wasting away, my internal organs were failing one by one, I was needing ever more invasive interventions until 2011 when we received the news that my condition would drastically shorten my lifespan. I received the news in 2012 that I wasn't expected to live another 5 years (Watts 2017, np).

As a young disabled person, no one has asked me whether I'm scared about my future or whether my life‐limiting condition has impacted my life choices. These are not pleasant things to think about, but I can promise you, nearly every disabled person has thought about them (Vogelmann, quoted on the project website^6^).

One can read our co‐researchers reactions to life‐limiting impairments as understandable affective responses to shortened lives. Disabled bodies and minds – particularly those that are living short lives – are wrapped up in dominant tragedy discourses and these ideas trickle down into how disabled people and non‐disabled people internalise disability. Co‐researchers routinely talk to us as much about pain and suffering as they do more affective responses as to what it means to live a short life, and the emotional consequences of these: leaving loved ones; guilt/feeling burdensome; and ‘missing out’ and routine exclusion. Here the work of writers such as Marks (1999), Thomas (1997) and Reeve (2002) articulate this as internalised oppression: where psyches, psychologies and subjectivities are engulfed by pathological understandings of being disabled. Clearly, our co‐researchers do not shirk from the realities or constructions associated with life‐limiting impairments. They are very much aware of the mourning trope that underpins many constructions and experiences of living with LL/LTIs. And their accounts work the humanist register: raising questions about how one might live a life that is short or painful. But, again, this is not the only story; nor is it the only philosophy that they are living with. Take for example the following commentary provided by Lucy:

I'm 23‐years‐old; an age, if I went by my prognosis, which I should never have reached. I have a life‐limiting condition. Yet, my life is pretty amazing. However, if I told you the reason my life is so great is because of my condition, would you believe it? (Watts 2017, np).

Lucy's account short‐circuits the usual discourses of melancholia and mourning. Lucy is keen to define her legacy, to be remembered and leave her mark on the world. There is, we would argue, a more affirmative account being developed here. Braidotti (2010: 44) argues that critical and social theory is founded upon a negative melancholia. She argues:

There is an implicit assumption that political subjectivity or agency is about resistance and that resistance means the negation of the negativity of the present. A positive is supposed to be engendered by this double negative. Being against implies a belligerent act of negation, erasure of present conditions.

Such a politics resonates with the humanist politics of disability that seeks answers to the barriers that disabled people face, contests negative discourses of disability‐as‐tragedy and engages with the realities of impairment. But Lucy's assertion that ‘the reason my life is so great is because of my condition’ invites a different philosophical and activist position, one that Braidotti (2010: 44) defines as ‘a non‐human, vitalistic and affirmative dimension of subjectivity’. This is an affirmative project that stresses positivity and not mourning’; ‘in terms of an ethics of affirmation’ there is ‘also an ethology of forces’ (Braidotti 2010: 45). These driving forces concretise in actual, material relations and can thus constitute a network, web or rhizome of interconnection with others. We have to learn to think differently about ourselves. To think means to create new concepts’. This segues well with further words from Lucy:

It was doing end of life planning in 2011 that led to me having a whole new life. An exciting, purposeful, enjoyable life, in spite of my deteriorating condition and all its complications and the limits these impose on my life. I would start to truly ‘live’ again and have a life to be proud of… Since that day, I've achieved an awful lot. I've written blogs and articles for various places; written forewords to three guidances and a book; I've delivered many speeches at events from national conferences to meetings to informal groups; I've appeared on television and radio and in the media; I'm a project advisor on a research project; I manage the website and social media for one charity; and more. I hold positions within six charities and one alliance, and work with many other charities and organisations on a one‐off intermittent basis (Watts 2017, np).

Braidotti (2010: 47) proposes an affirmative ethics for a non‐unitary subject that proposes an enlarged sense of inter‐connection between self and others, including the non‐human or ‘earth’ others. This practice of relating to others requires and is enhanced by the rejection of self‐centred individualism. It implies a new way of combining self‐interests with the wellbeing of an enlarged sense of community, which includes one's territorial or inhuman, i.e. environmental inter‐connections. It is an eco‐philosophy of multiple belongings for subjects constituted in and by multiplicity. Lucy appears, then, to straddle humanist and posthumanist politics:

I've gained a purpose, a new life and its led me to truly live. I am no longer merely existing, I now have a joyful, exciting, jam‐packed life. I still have a degenerative and life‐limiting condition, I'm continuously getting worse and losing abilities, my condition becomes more fragile and harder to manage, I require more intervention and I have to deal with the almost continuous cycle of grief with each new problem, complication and deterioration, but I have a life that is worth living. A life I'm proud of. Had I not been poorly, I'd have recently qualified as a junior doctor, which has been a cruel blow, but I am regularly told that I've likely done more with my life and made a greater contribution to society as a patient leader and ambassador than I ever would have as a doctor. I like to think that is true (Watts 2017, np).

Lucy encourages us to re‐evaluate our own plans and desires in relation to the ending of lives. Here, yet again, we are left with a clear sense of the complex philosophical and political work undertaken by our co‐researchers.

## Conclusion: DisHuman conditions

Reflecting on the significant impact of our co‐researchers’ writing we think that our collaborative work together contributes to two areas of theoretical development. Firstly, our paper adds to a new interdisciplinary field of scholarship entitled the Critical Posthumanities (Braidotti 2018). In this piece Braidotti acknowledges disability studies as a ‘crucial generative trans‐disciplinary hub of posthuman knowledge’ that is ‘firmly grounded in the present (as actual and virtual), which means that they take real‐life events seriously, and by extension, take power seriously’ (Braidotti 2018: 9). Critical Posthumanities is ‘The Missing Peoples’ Humanities’ (Braidotti 2018: 19). Studies such as these critique humanism, expand upon posthuman possibilities and, at times, offer ‘alternative visions of the humanist, knowledge and society’ (Ibid). Indeed, as our analyses have shown, our co‐researchers posit critical understandings of posthuman and humanist lives. They are, at the very mundane, living the practicalities of a Critical Posthumanities. Secondly, our paper contributes further to our work associate with a DisHuman perspective that we understand as contributing to the development of the posthumanities (see Goodley *et al*. 2014, 2015, Goodley 2016, Liddiard *et al*. 2018). Our work centres around a key question:

What does it mean to be human in the 21st Century and in what ways does disability enhance these meanings?… We also ponder what it might mean to be *DisHuman;* ways of being in the world where disability illuminates a moment of reflection for humanity, and contests deep‐rooted productions of the archetypal human and neoliberal citizen as self‐contained, autonomous, independent, strong and self‐governing’ (dishuman.com)

A DisHuman perspective disavows the humanist human: we are drawn to its usage (especially when disabled people use humanism as a framing for recognition) and, equally, are repulsed by humanism's exclusionary nature (hence pushing us into more posthuman territories). This generative scholarship is mirrored in the contributions of our co‐researchers, who we would understand as DisHuman theoreticians in their own right. As Sally reflects on her life now, happily married to Ed and with her Canine Partner Ethan:

Now, almost a year on, I'm the happiest I've ever been. I love that he [Ed] doesn't see my failing body or my own self‐perceived brokenness – he just sees me. And Ethan – not a day goes by where I don't marvel at the extraordinary work he does for me and his unconditional love (Watts, 2018).

Sally does not dismiss her disabled identity but does want to be recognised as a humanist self that, in our disabling culture, risks being erased by the presence of disabling practices. We also read of the equal significance of Ethan in her life. Thus, humans, machines and animals populate her DisHuman positionality: where she claims recognition of the sovereign self alongside a celebration of the posthuman assemblage of body, wheelchair and assistance dog. Similarly, Lucy reports on her assistance dog Molly:

Molly is also an Internet star, blogger (see http://mollydogwithablog.blogspot.com/), friend and comrade to Lucy, and part‐time Cocker Spaniel. In 2016, she was awarded an animal version of an MBE, an Order of Merit awarded medal that is ‘awarded to recognise animals that have shown outstanding acts of devotion and that symbolise the special relationship between animals and humans’ (PDSA 2016, np) (Watts and Liddiard 2017, np).

To recognise animals through an Order of Merit might be read as a deeply anthropocentric act. This contradictory DisHuman position captures the straddling of humanist and posthuman positions occupied by Lucy and Molly. Their assemblage is a truly posthuman one and one, in this case, valued too by a humanist cultural imaginary. Lucy and Molly preempt Braidotti's recent writing; specifically her argument that we are all now ‘humanimals’, trans‐corporeal human‐animal compounds (Braidotti 2018; 10) but we are also clearly engaging at the same time with neohumanist claims (Braidotti 2018: 4). As Sally commented in a written response to the paper:

I would go so far as to say that Ethan has taught me how to be a person better. He has showed me what total acceptance is regardless of ability or disability. He has taught me how to live a life that is more in the present and unburdened by worries for the future or shortened life.

Our co‐researchers are ‘posthuman subjects of knowledge – ‘embedded, embodied and yet flowing in a web of relations with human and non‐human others’ (Braidotti 2018: 4). We want to take a moment to anticipate one, of the many, perhaps, criticisms of our approach. By co‐creating an alternative analysis of the lives of young people with life‐limiting impairments which focuses on their vibrant and full lives, we could be accused of trying to replace one stereotypical account of disability with another (Watermayer, 2013). And yet, we would argue that our DisHuman orientation enables us to resist stereotypes and to acknowledge that the web of relations between humans and others means that our subject positions are always moving and always in flux. Clearly, our co‐researchers are involved in some complex activist and theoretical work and we can conclude with confidence that their lives are anything other than limited.

## Notes

1
https://livinglifetothefullest.org/

2
https://livinglifetothefullest.org/2018/04/19/co-researcher-voices-speaking-out/

3
https://livinglifetothefullest.org/2017/09/28/planning-for-the-end-of-my-life-aged-just-17-made-me-live-a-life-that-ensured-i-wasnt-forgotten/

4
https://livinglifetothefullest.org/2018/02/08/exploring-the-world-side-by-side/

5 Symposium hosted by the Institute for the Study of the Human (iHuman), *Animal‐Machine‐Human: The place of disability*, held on 10th October 2017 at the University of Sheffield

6
https://livinglifetothefullest.org/the-co-researcher-collective/
